# Analysis of EBNA-1 and LMP-1 variants in diseases associated with EBV infection in Chinese children

**DOI:** 10.1186/1743-422X-9-13

**Published:** 2012-01-11

**Authors:** Junhong Ai, Zhengde Xie, Chunyan Liu, Zhizuo Huang, Junmei Xu

**Affiliations:** 1 Key Laboratory of Major Diseases in Children and National Key Discipline of Pediatrics (Capital Medical University), Ministry of Education, Beijing Pediatric Research Institute, Beijing Children's Hospital, Capital Medical University, Beijing 100045, China

**Keywords:** EBV, EBNA-1, LMP-1, variant, children

## Abstract

**Background:**

In China, primary EBV infection occurs during childhood with seroprevalence reaching about 100% by 10 years of age. There are few studies on EBV variants in diseases associated with EBV infection in Chinese children. In this study, we investigated the diversity of the EBV genes (EBNA-1 and LMP-1) and the relationship between EBV variants and the clinical phenotypes in diseases associated with EBV infections in Chinese pediatric cases.

**Results:**

The frequencies of EBV type I in the IM, HLH and HL samples were 98.4%, 100% and 95.8%, respectively. Three known EBNA-1 variants were identified, including V-val (all were V-val-v1 sub-variant), P-thr' and V-Leu (MT). The frequency of V-val-v1 was 98.6% in the IM samples, 100% in the HLH samples and 97.1% in the HL samples. There were no significant differences of the distribution of EBNA-1 variants between IM, HLH and HL samples (P > 0.05). Three known LMP-1 variants, including China 1, China 2 and Med, were identified and China 1 was predominant in all groups (IM 88.6%, HLH 100% and HL 100%). The frequency of del-LMP-1 was 88.6% in the IM samples, 100% in the HLH samples and 96.0% in the HL samples. There were no significant differences in the frequency of del-LMP-1 between the IM, HLH and HL samples (P > 0.05). The frequency of *Xho*I loss was 90.6% in the IM samples, 100% in the HLH samples and 100% in the HL samples, with no significant difference in frequency (P > 0.05). In the EBV type I strain, V-val-v1 variant (EBNA-1) was linked with China1 variant (LMP-1) in 88.9% of the IM samples, 100% of the HLH samples and 80.0% of the HL samples in this study.

**Conclusions:**

Type I EBV was the most prevalent subtype EBV in Chinese pediatric cases and V-val-v1 (EBNA-1) and China1 (LMP-1) variants were the most dominant variants. There was a strong linkage between V-val-v1 (EBNA-1) variant and China1 (LMP-1) variant in type I EBV. The sequence variation in EBV genes may represent a geographic polymorphism since no preferential associations were found between specific EBV variants and specific diseases in this study.

## Background

Epstein-Barr virus (EBV) is a lymphotrophic human gamma-1 herpes virus with a double stranded DNA genome comprised of approximately 170-kilobases. It is transmitted primarily through saliva and infects over 95% of the world's population [1]. In developing countries, primary EBV infection typically occurs in early childhood and is asymptomatic; in developed countries, infection occurs in later childhood or young adulthood and can manifest as infectious mononucleosis (IM), which is self-limiting. EBV is associated with not only nonmalignant diseases but also a number of malignant diseases, including Burkitt's lymphoma, nasopharyngeal carcinoma and Hodgkin lymphoma.

After primary infection, EBV establishes a lifelong latent infection in B lymphocytes [2]. During latent infection, EBV expresses a restricted set of genes, including two EBV-encoded RNAs (EBER-1 and EBER-2), six EBV nuclear antigens (EBNA-1, EBNA-2, EBNA-3A, -3B, -3C, and leader protein EBNA-LP), and three integral membrane proteins (LMP-1, LMP-2A and -2B).

Based on the sequence divergence of nuclear antigens (EBNA-2, -3A, -3B, and -3C) EBV can be divided into two types of EBV: type I and type II [3,4]. Detailed sequence analysis has also been done with the EBNA-1, LMP-1 and BZLF1-genes, which are not type-specific in their sequence divergence among EBV strains.

EBNA-1 sequence variation has been described by comparing the sequence of several EBV isolates from tumors and cell lines with the sequence of prototype B95-8 [5]. EBV has been classified into five EBNA-1 variants based on polymorphism of the C-terminal region and the signature changes at amino acid residue 487 of EBNA-1, including the prototype P-ala and P-thr variants and the V-val, V-leu and V-pro variants [6].

LMP-1 gene polymorphisms were identified in several studies [7-11]. The most common polymorphisms contain substitutions of the nucleotides or amino acids including loss of restriction sites (such as *Xho*I in the N-terminus), variations of the number of 33 bp repeats, and a 30 bp deletion in the C-terminus (del-LMP-1). Ten major variants of LMP-1 (China 1, China 2, China 3, NC (North Carolina), Mediterranean+ (Med+), Med-, GD1, SEA 1, and SEA 2) have been described from different regions and diseases [8-10].

In China, primary EBV infection occurs during childhood with seroprevalence reaching about 100% by 10 years of age. However there are few studies on EBV variants in diseases associated with EBV infection in Chinese pediatric population [12]. So in this study, we investigated the diversity of the EBV genes EBNA-1 and LMP-1 in IM (a self-limiting disease), EBV associated Hemophagocytic lymphohistiocytosis (EBV-HLH) (a severe hematological disease triggered by EBV) and Hodgkin lymphoma (HL) in Chinese children.

## Results

### EBV types I and II in EBV associated diseases in children

The frequency of EBV type I or type II infection was determined in 107 samples. Type I EBV was present in 105 samples (98.1%, 105/107) and type II EBV in 2 samples (1.9%, 2/107) (Table 1).

**Table 1:** Distribution of EBV subtypes in IM, HLH and HL patients

EBV subtypes	IM (n = 61)	HLH (n = 22)	HL (n = 24)
Type I	60 (98.4%)	22 (100%)	23 (95.8%)
Type II	1 (1.6%)	0	1 (4.2%)

### EBNA-1 variants in EBV associated diseases in children

The C-terminal EBNA-1 fragment was amplified from 128 samples (Table 2). In this study, three known variants of the EBNA-1 including V-val (all were V-val-v1 Sub-variant), P-thr' and V-Leu (MT) [13-15] were observed (Table 2). Sub-variant V-val-v1 of EBNA-1 first reported by Do et.al [13] was the most common in the three groups (IM 98.6%, HLH 100%, HL 97.1%). There were no significant differences in the incidence of EBNA-1 variants between the IM, HLH and HL samples (P > 0.05).

**Table 2:** Distribution of EBNA-1 variants in IM, HLH and HL patients

variants	IM (n = 71)	HLH (n = 23)	HL (n = 34)
V-val-v1	70(98.6%)	23 (100%)	33 (97.1%)
P-thr'	1(1.4%)	0	0
V-Leu(MT)	0	0	1 (2.9%)

### LMP-1 variants in EBV associated diseases in children

**Figure 1:**
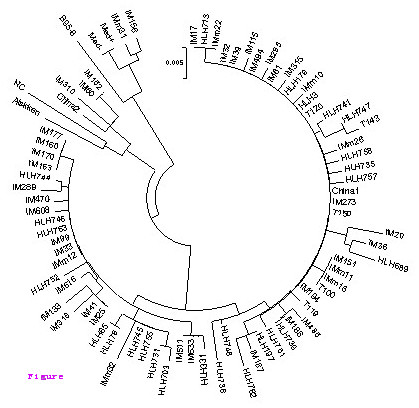
**Phylogenetic tree of the C-terminus of LMP-1**. B95-8 variant according to Genebank: V01555.2. China 1 (Genebank: AY337723.1), China 2 (Genebank: AY337724.1), Alaskan (Genebank: AY337725.1), Med- (Genebank: AY337721.2), Med+ (Genebank: AY337722.2) and North Carolina (NC) (Genebank: AY337726.2) are the LMP-1 variants according to Miller et al.(1994), Sung et al.(1998) and Edwards et al (1999). IM: infectious mononucleosis; HLH: hemophagocytic lymphohistiocytosis. 'm' indicated the outpatient and the figure means the mumber of the sample.

A total of 74 LMP-1 nucleotide sequences corresponding to the carboxyl-terminal were obtained and translated into amino acid for the phylogenetic analysis (Figure 1). Three known variants of LMP-1 including China 1, China 2 and Med were identified (Table 3). Among them China 1 was predominant in all groups. The frequency of China 1 in the IM, HLH and HL samples was 88.6% (39/44), 100% (25/25) and 100% (5/5), respectively.

**Table 3:** Distribution of LMP-1 variants in IM, HLH and HL patients

variants	IM (n = 44)	HLH (n = 25)	HL (n = 5)
China1	39 (88.6%)	25 (100%)	5 (100%)
China2	3 (6.8%)	0	0
Med	2 (4.6%)	0	0

A short fragment of LMP-1 containing the 30 bp deletion region was amplified from 20 paraffin-embedded tumors biopsies. Nineteen of them contained the del-LMP-1, and 1 specimen had wild type LMP-1 (wt-LMP-1). In total, the del-LMP-1 was detected in 88.6% (39/44) of IM samples, 100% (25/25) HLH samples and 96.0% (24/25) HL samples. There were no significant differences of the incidence of del-LMP-1 in three groups (P > 0.05).

The full length of LMP-1 was amplified in 54 samples. LMP-1 variant with *Xho*I loss was detected in 90.6% (29/32) of the IM, 100% (17/17) of the HLH and 100% (5/5) of the HL samples, respectively. There was no significant difference in the frequency of *Xho*I loss between the IM and HLH samples, or between the IM and HL samples (P > 0.05).

### Linkage analysis

A combination of more than one polymorphic site in the EBV genome was investigated in 60 samples for this study. All 60 samples were EBV type I strains. The frequency of V-val-v1 (EBNA-1) variant lingking China1 (LMP-1) variant in the IM, HLH and HL samples was 88.9% (32/36), 100% (19/19) and 80.0% (4/5), respectively (Table 4). Preferential linkages of certain EBNA-1 variants to distinct LMP-1 diversity were found to exist. However, there were no significant differences between the distribution of linked EBV genes in the IM, HLH and HL samples (P > 0.05).

**Table 4:** Linkage analysis of EBV type, EBNA-1 and LMP-1 variants

EBV type	EBNA-1 variant	LMP-1 variant	Number of Cases (%)		
			
			IM	HLH	HL
Type I (60)	V-val-v1	China1	32 (88.9%)	19 (100%)	4 (80.0%)
		China2	2 (5.6%)	0	0
		Med	1 (2.8%)	0	0
	P-Thr'	China1	1 (2.8%)	0	0
	V-Leu(MT)	China1	0	0	1 (20.0%)
Type2 (0)	0	0	0	0	0

## Discussion

EBV is a lymphotrophic human gamma-1 herpes virus. It is classified as EBV types I and II based on sequence variation in the EBNA2 and EBNA3 genes [3,4]. Previous studies of healthy Asian populations showed that 85% of individuals were infected with EBV type I, 4% with EBV type II and 11% with both types [16-18]. In this study, the frequencies of EBV type I in IM, HLH and HL were 98.4%, 100% and 95.8%, respectively. No co-infection with both viral types was detected. This difference can be explained by the small number of samples analyzed in this work, or alternatively, that pediatric patients are still not subjected to subsequent re-infections as adults [1].

EBNA-1 is the only latent protein that is consistently expressed in all EBV-positive malignancies. EBNA-1 is critical in maintaining EBV in the infected cells and facilitating episomal replication [19,20]. In replicating latently infected EBV cells, EBNA-1 dictates equal partitioning of EBV genome, thus reducing the loss of EBV infected cells [21]. EBNA-1 is also a transcriptional activator and activates expression of EBV transcripts via the latent C promoter [22]. EBNA-1 carboxy (C)-terminal sequence variants have been described based on the amino acid signature at codon 487, and designated prototype including P-ala (identical to prototype B95.8 strain), P-thr, and variant including V-val, V-leu, and V-pro [6]. New variants and subvariants have more recently been characterized [1,13,23].

In Asian, most studies of the EBNA-1 variations have focused on nasopharyngeal carcinoma (NPC). Mai et al. suggested that V-val EBNA-1 with the functional advantage compared with prototype shown in their study might contribute to the tumorigenesis of NPC by increasing the expression of itself or other viral or cellular genes [24]. Zhang et al. indicate that V-val variant infects NPC preferentially and that a susceptibility to a particular EBV isolate in the nasopharynx may exist during development of NPC [25]. However, Sandvej et al. found that V-val is the dominant variant in Asian regions and appear to be associated with Asian NPC patients but not found in Danish NPC biopsies [23]. Do et al. reported that V-val-v1 (EBNA-1) is predominant in NPC as well as in healthy EBV-carriers from an Asian population [13]. Wang et al. observed that V-val was the most common variant not only in NPC but also in EBV associated gastric carcinoma and healthy donors in Northern China [26]. Moreover, a review about genetic diversity of EBV showed that V-val was detected in both cases and controls almost exclusively in China [27].

IM is a self-limiting disease caused by primary EBV infection. EBV-HLH is a severe hematological disease triggered by EBV infection and HL is a malignant disease. In this study, the percentage of V-val-v1 (EBNA-1) in IM, HLH and HL were 98.6%, 100% and 97.1%, respectively. No significant differences were found in the incidence of V-val-v1 subvariant in IM, HLH and HL groups (P > 0.05). The results indicated that the V-val-v1 is the dominant variant in Chinese pediatric cases, and EBNA-1 polymorphism may represent a geographic polymorphism. The data from Argentinean pediatric population showed that V-leu sub-variants preferentially circulate in this region. Interestingly, they also favor the geographical association hypothesis since no evidence for a preferential compartment distribution of EBNA-1 variants and sub-variants was found [1].

LMP-1 is considered the EBV oncogene [28]. It encompasses a 25 aa N-terminus, six predicted transmembrane domains (aa: 26-196), and a C-terminus (aa: 197-386). Both the N- and C-termini are located in the cytoplasm of the cell [29,30]. The C-terminus interacts with cellular proteins through the C-Terminal Activation Region 1 (CTAR1) and CTAR2 and activates several signaling pathways, including the transcriptional nuclear factor-kB (NF-kB) whose activation is linked to the inhibition of apoptosis [31,32]. The most commonly reported LMP-1 gene polymorphisms was a 30 bp deletion in the C-terminus. The 30 bp deletion variant (del-LMP-1) was first detected in EBV isolated from cell lines derived from NPC patients from Southern China [33]. China 1 and Med+ variants have this 30 bp deletion.

A number of reports have focused on the del-LMP-1 variant in malignant diseases. Hadhri et al. found that del-LMP-1 variant was significantly (P = 0.006) more frequent in NPC (71.42%) than in control biopsies (52%) in Tunisia [34]. Tiwawech et al. also reported that a significant association between the del-LMP-1 variant and NPC susceptibility (P = 0.023) was exist in Thais. Moreover, the frequency of del-LMP-1 in NPC patients was associated with the clinical stage of NPC [16]. Research from South America (35 Brazilian HD patients and 27 Argentine pediatric patients of HD) show that del-LMP-1 was more frequently detected in Hodgkin's lymphoma than in non-neoplastic controls (P = 0.005) suggesting a pathogenic role of del-LMP-1 variant in Hodgkin's lymphoma in this region [35]. These results supporting the idea that EBV del-LMP-1 may be a disease-associated variant.

However, there were some controversial findings. See et al. concluded that the presence of del-LMP-1 was associated with the Chinese race [36]. Saechan et al. found a higher prevalence (P = 0.026) of del-LMP-1 variant in Southern Thais than in Central Thais [9]. In this study, there was no significant difference in the frequency of del-LMP-1 between the IM, HLH and HL samples (P > 0.05) in Chinese children. And the same trend was also observed in another study in China, with 83% of Hodgkin's lymphoma and 86% of healthy controls showing del-LMP-1 variant [37]. Thus, the del-LMP-1 variant may represent a geographic or race associated polymorphism.

The loss of *Xho*I restriction site in the N-terminus of the LMP-1 gene is another commonly reported LMP-1 gene polymorphism. Hu et al. first described the loss of the *Xho*I site from exon 1 of the LMP-1 gene in the CAO cell line derived from a Chinese NPC case [28]. The loss of the *Xho*I site is caused by a single base substitution from G to T at the nucleotide position 169425. It has been reported that the *Xho*I loss was significantly associated with Asian NPC patients and the *Xho*I loss has been considered as a specific HL marker [28,38,39]. However, See et al. found that the *Xho*I loss was not only associated with type III NPC, but also with the Chinese race [36]. In this study the frequency of *Xho*I loss was 90.6% in the IM samples, 100% in the HLH samples and 100% in the HL samples. There was no significant difference in the frequency of *Xho*I loss between the IM and HLH samples or the IM and HL samples (P > 0.05). These findings suggest that the loss of *Xho*I site may not be a polymorphism associated with disease phenotypes.

Studies conducted to date have focused on EBV within different polymorphic regions, making comparisons across studies difficult and limiting the ability to define the full spectrum of diversity within the EBV genome [27]. Thus, a combination of more than one polymorphic site in the EBV genome might be involved in determining disease characteristics. This study showed a high linkage between V-val-v1 variant (EBNA-1) and China1 variant (LMP-1) in type I EBV strains. There were no significant differences between the distribution of the linkage in the IM, HLH and HL samples (P > 0.05).

There were some limitations of this study. Because of relatively small size of samples, some variants, such as P-thr, was detected with very low positive rate compared with previous study in this region. Some blood samples may harbor a lower level of viral load, and PCR products were not obtained from all IM and HLH samples.

## Conclusions

In conclusion, this study described the diversity of the EBV genes and investigated the association between EBV genotypes and the clinical phenotypes of EBV-related diseases in Chinese pediatric cases. Type I EBV was the most prevalent subtype EBV in Chinese pediatric cases and V-val-v1 (EBNA-1) and China1 (LMP-1) variants were the most dominant variants. There was a strong linkage between V-val-v1 variant (EBNA-1) and China1 variant (LMP-1) in type I EBV. The sequence variation in EBV genes may represent a geographic polymorphism since no preferential associations were found between specific EBV variants and specific diseases. A more comprehensive exploration of EBV diversity across its entire genome might allow for the development of a consensus classification system of EBV variants into variant classes that could guide subsequent studies aimed at understanding EBV geographic and disease associations [27].

## Methods

### Patients and Samples

One hundrard and sixteen peripheral blood specimens were obtained from pediatric patients in Beijing Children's Hospital Affiliated Capital Medical University. Eighty four were IM and 32 were EBV-HLH. IM and HLH were diagnosed according to their respective diagnostic criteria [40,41]. All procedures were reviewed and approved by the Committee of Human Studies at the Beijing Children's Hospital affiliated the Capital Medical University. A written informed consent was obtained from all patients' parents.

Thirty four paraffin-embedded tissue samples and five fresh tissue samples from HL cases were also enrolled into this study.

### DNA extraction

Genomic DNA was extracted from 2 ml whole blood samples by using a blood genomic DNA isolation kit (Cat#RT403, Tiangen, China) according to manufacturer's instructions. A QIAamp DNA FFPE Tissue kit (Cat.No.56404, QIAGEN GmbH, Hilden, Germany) was used to extract the DNA from the paraffin-embedded HL tissues following manufacturer's instructions. The isolation of DNA from the fresh HL biopsies was performed using a phenol-chloroform extraction method.

### PCR amplification and sequence analysis

#### Definition of EBV type I/II

EBV type was determined by PCR with specific primers for EBNA3C. The sequences and positions of these primers are as follows: EBNA3C-F, 5'-AGA AGG GGA GCG TGT GTT GT-3' (B95-8 coordinate 87651-87670); EBNA3C-R, 5'-GGC TCG TTT TTG ACG TCG GC-3' (B95-8 coordinate 87803-87784), which yield an amplification product of 153 bp for EBV-I and a product of 246 bp for EBV-II, as previously described [3]. PCR was performed in 50 ul using 1.5 ul of 10 pM of the forward and reverse primers, 25 ul 2 × Hotsart Taq PCR Master Mix (Tiangen, China), 5 ul genomic DNA. The reaction mixture was initially denatured at 95°C for 5 min followed by 35 cycles including denaturation at 95°C for 45 s, annealing at 56°C for 45 s, extension at 72°C for 1 min, and finally elongation at 72°C for 10 min. After PCR assay, the amplified products were subjected to electrophoresis in 2% agarose gel and visualized under ultraviolet light.

#### EBNA-1 amplification

EBNA-1 C-ter region was amplified by nested PCR. The primers were from a previous study [1]. PCR was performed in 50 ul contained 1.5 ul of 10 pM of the forward and reverse primers, 25 ul 2 × Hotsart Taq PCR Master Mix (Tiangen, China), and 1.5 ul genomic DNA. PCR conditions were the same to the previous study [1]. The amplified fragments were 329 bp.

#### LMP-1 amplification

The oligonucleotide primers were designed to amplify the full length of LMP-1. The sequences and positions of these primers are as follows: LMP-1-F1, 5'-AGG GAG TGT GTG CCA GTT AAG-3' (B95-8 coordinate 168053-168073); LMP-1-R1, 5'-CAA ACA CAC GCT TTC TAC TTC C-3' (B95-8 coordinate 169679-169700); LMP-1-FS1, 5'-AGG TTA GTC ATA GTA GCT TAG CTG-3' (B95-8 coordinate 168157-168180); LMP-1-RS1, 5'-TCA ACG CAG TCT TAG GTA TCT G-3' (B95-8 coordinate 168953-168974); LMP-1-FS2, 5'-AGG GAG TCA TCG TGG TGG TGT-3' (B95-8 coordinate 168917-168739); LMP-1-RS2, 5'-ACT GCC CTG AGG ATG GAA CAC-3' (B95-8 coordinate 169466-169486).

The first round polymerase chain reaction (PCR) was performed with LMP-1-F1 and LMP-1-R1. 1 ul of 10 pM of the forward and reverse primers, 12.5 ul 2 × Hotsart Taq PCR Master Mix (Tiangen, China), and 2 ul genomic DNA were mixed in a 25 ul reaction. The reaction mixture was initially denatured at 94°C for 5 min followed by 30 cycles including denaturation at 94°C for 45 s, annealing at 53°C for 45 s, extension at 72°C for 90 s, and finally elongation at 72°C for 5 min. The products of the first round PCR were amplified in the second round PCR with LMP-1-FS1 and LMP-1-RS2 or with LMP-1-FS1, LMP-1-RS1 and LMP-1-FS2, LMP-1-RS2 as described above. In the second round PCR the reaction volume was increased to 50 ul. The amplified fragments were 1648 and 1330 (or 818 and 786) bp, respectively.

For the paraffin-embedded HL tissues, a short fragment of LMP-1 containing the 30 bp deletion region was amplified also using the nested PCR. The sequences and positions of the primers are as follows: del-LMP-1F1, 5'-CTA GCG ACT CTG CTG GAA AT-3' (B95-8 coordinate 167934-167915); del-LMP-1R1, 5'-GAG TGT GTG CCA GTT AAG GT-3' (B95-8 coordinate 167598-167617); del-LMP-1F2, 5'- TGG AGG GAG AGT CAG TCA GGC-3' (B95-8 coordinate 167643-167663); del-LMP-1R2, 5'-ATT GAC GGA AGA GGT TGA AAA-3' (B95-8 coordinate 167897-167877). The first round polymerase chain reaction (PCR) was performed with del-LMP-1F1 and del-LMP-1R1. 1 ul of 10 pM of the forward and reverse primers, 12.5 ul 2 × Hotsart Taq PCR Master Mix (Tiangen, China), and 6 ul genomic DNA were mixed in a 25 ul reaction. The reaction mixture was initially denatured at 95°C for 5 min followed by 30 cycles including denaturation at 94°C for 45 s, annealing at 56°C for 45 s, extension at 72°C for 1 min, and finally elongation at 72°C for 10 min. The products of the first round PCR were amplified in the second round PCR with del-LMP-1F2 and del-LMP-1R2. In the second round PCR the reaction volume was increased to 50 ul and the genomic DNA was decreased to 4 ul. The amplified fragments were 337 and 255 bp, respectively.

### Sequence analysis

Sequence analysis was performed on an ABI3730XL Genetic Analyzer. Sequences were aligned and analyzed with Bioedit V7.0.9 software. Sequences were compared with B95.8 reference sequence (GenBank V01555) and with other isolates from GenBank database. Phylogenetic tree were made by MEGA V4.0.2 software.

### Statistical analysis

SPSS 13.0 for windows was used for statistical elaboration. A Chi-square test or a Fisher's exact test was used to compare the distribution of EBV type I and II, EBNA-1 and LMP-1 variants between the IM, HLH and HL samples. *P*-values less than 0.05 were considered to be significant differences.

## Competing interests

The authors declare that they have no competing interests.

## Authors' contributions

JHA carried out most of the experiments and wrote the manuscript. CYL, ZZH and JMX helped with the experiments. ZDX designed the experiments and revised the manuscript. All of the authors read and approved the final version of this manuscript.
